# The Impact of Interaction Time and Verbal Engagement with Personal Voice Assistants on Alleviating Loneliness among Older Adults: An Exploratory Study

**DOI:** 10.3390/ijerph21010100

**Published:** 2024-01-16

**Authors:** Changmin Yan, Kate Johnson, Valerie K. Jones

**Affiliations:** College of Journalism and Mass Communications, University of Nebraska-Lincoln, Lincoln, NE 68588, USA; kjohnson158@huskers.unl.edu (K.J.); valerie@unl.edu (V.K.J.)

**Keywords:** aging adults, loneliness, Amazon Alexa, quantity of speech, older adults, loneliness intervention, personal voice assistant, gerontology, human–computer interaction

## Abstract

Background: Loneliness in older adults is a critical issue that negatively affects their well-being. The potential of personal voice assistant (PVA) devices like Amazon’s Alexa Echo in reducing loneliness is an emerging area of interest, but it remains under-researched. Objective: this study aims to investigate the effect of interaction time and verbal engagement with PVA devices on reducing loneliness among older adults living alone. Method: In this experiment, individuals aged 75 and older (*n* = 15), living alone, were provided with Amazon Alexa Echo devices. They were instructed to interact with the device at least five times a day for a duration of four weeks. The study measured participants’ loneliness levels using the UCLA loneliness scale both before and after the study. Additionally, the interaction time and verbal engagement with the device were measured by the total time of use and the total number of intentional commands spoken to Alexa during the four-week period. Results: The findings revealed that the total time spent interacting with Alexa was a significant predictor of loneliness reduction. A mediation analysis indicated an indirect effect, showing that the number of intentional commands spoken to Alexa contributed to loneliness reduction indirectly by increasing the total time spent with the device (verbal engagement → interaction time → loneliness reduction). Conclusions: This study suggests that the key to reducing loneliness among older adults through PVA devices is not just initiating verbal interaction, but the overall time devoted to these interactions. While speaking to Alexa is a starting point, it is the duration of engagement that primarily drives loneliness alleviation.

## 1. Introduction

Loneliness, a complex aspect of the human experience, is marked by feelings of distress and unfulfillment due to perceived unmet social needs, particularly in the quality and quantity of social relationships [1,2,3,4]. This phenomenon is often accompanied by emotional discomfort and social isolation [5,6,7].

Older adults, especially those above 75, are disproportionately affected by loneliness. Studies highlight a significant increase in self-reported loneliness in this age group [8,9,10]. Research in Amsterdam shows a positive correlation between age and loneliness, with 40–50% of those over 80 experiencing moderate to severe loneliness [11]. Similar findings emerge from studies in Sweden [12], China [13], Finland [14], and Canada [15]. The impact of COVID-19 on increasing loneliness among older people is well-documented [16,17]. Loneliness in this demographic is linked to depression and increased mortality risks [18,19], emphasizing the societal importance of addressing loneliness among the rapidly growing global population of older adults [20].

Personal voice assistant (PVA) devices (also referred to as “intelligent personal assistants” [21] or just “voice assistants” [22]) are devices that listen to, interpret, and respond to speech using dialogue systems to analyze voice and language [23]. PVAs can answer a diverse array of questions and perform various tasks. Users can inquire about general information, request navigation directions, set reminders, initiate web searches, engage in entertainment and fun interactions, manage productivity, control smart home devices, monitor health and wellness, and engage in personalized and conversational dialogues [24]. PVAs, such as Siri, Google Assistant, and Alexa, offer versatile and convenient assistance across a wide spectrum of daily activities and needs. PVA devices are emerging ICT interventions showing potential for reducing loneliness among older adults [25,26,27,28]. These hands-free devices, which offer functionalities like music, reminders, and verbal companionship [29,30], are particularly beneficial for their companionship aspect and inclusivity for those with physical limitations [31,32]. In a scoping review of 18 published studies, assistive technologies such as memory aids and health tracking devices seem to be effective in reducing loneliness and social isolation among older adults [33]. Such effects could in part be explained by lonelier people’s predisposition to accept PVA. In an online survey study, participants perceived voice AI devices as socially attractive and had fewer privacy concerns, which increased their satisfaction and likelihood of continued use [34].

The amount of time spent with a voice AI or assistive technologies could play a moderating role in engendering intended positive health and behavioral outcomes. Compared to a human voice, one-time exposure to an AI voice produced a higher level of fearful feelings and a lower level of perceived closeness to the AI speaker among the general population [35]. However, participants aged 50–90, when questioned about their long-term health conditions and general use of assistive technologies over time, revealed through survey and focus group data that utilizing assistive technologies had beneficial impacts on their health and social well-being [36]. This included enhancements in medication adherence, a rise in independence, and a decrease in loneliness.

In addition, the effectiveness of dialogue in loneliness reduction therapies [37] suggests that increased verbal interaction could reduce loneliness. An iPad-based video-call intervention using verbal interaction with family and friends reduced the perception of loneliness and increased perceived social connection among older people in care environments [38]. PVA devices are known to provide verbal companionship to older people [32] and could potentially replicate the same verbal interaction-based loneliness reduction effect when older people spend 3 weeks or longer with PVA devices [27].

This study investigates whether similar effects occur with extended PVA usage (i.e., 4 weeks), focusing on the relationship between loneliness reduction and two variables: time spent with the PVA and verbal engagement with the PVA. It explores a mediation pathway in which these variables potentially influence loneliness reduction, aiming to deepen our understanding of how PVA interactions might alleviate loneliness in older individuals. We therefore introduce the following hypotheses to systematically test the proposed relationships and pathways, thereby contributing to a more nuanced understanding of not only if but how older people–PVA interactions might mitigate loneliness:

**H1:**  *There will be a decrease in self-reported loneliness after the 4-week trial period.*

**H2:**  *There will be a positive correlation between the number of verbal engagement with a PVA device and a reduction in loneliness over the 4-week trial period.*

**H3:**  *Participants who spend more time interacting with their PVA device will report higher levels of loneliness reduction over the 4-week period.*

**H4:**  *There will be an “increase in verbal engagement → more interaction time → loneliness reduction” mediation path among participants over the 4-week period.*

## 2. Materials and Methods

### 2.1. Experimental Design

A pre–post PVA intervention study was designed to look at the relationships between PVA interactions and the reported loneliness of older adults, 70+, living alone. All protocols and materials were approved by the Institutional Review Board at the researchers’ university (IRB Number: 20190719327FB).

### 2.2. Recruitment, Screening, and Qualification of Participants

Participants from different cities in the Midwest region of the United States were recruited through flyers and information presentations at independent living facilities that consented to a partnership with the research team. Following presentations, interested older adults were screened to determine their eligibility for the study. During the screening session, participants were asked about their English fluency, age, living situation, ownership of a PVA, and were assessed using the Mini-Cog [39] and UCLA loneliness scale [40]. To be eligible, participants were required to be 75 years old or older, speak fluent English, live independently alone, not own an Amazon Echo or similar PVA, and not have a cognitive impairment. A total of 15 qualified participants were recruited to participate in a 4-week intervention study. Our sample size (*n* = 15) and study duration are consistent with previous studies on voice-activated virtual home assistants among adults 65 or older (average sample size = 16, sample size range: 7–19; study duration range: 3 weeks–12 months) [27,29,40,41].

### 2.3. Mini-Cog

The Mini-Cog analysis was used as a screening analysis to detect (not clinically diagnose) potential cognitive impairments or dementia in prospective participants. The Mini-Cog works on an algorithmic basis that correlates lower scores with an increased likelihood of cognitive impairment, and higher scores with a lower likelihood of cognitive impairment. For the purposes of this study, only individuals with normal cognitive functioning were eligible to participate [39].

### 2.4. Protocols

Following recruitment and screening, there were two subsequent in-person contacts with the participants: one visit, called session 1, on Day 1, for set-up, device instruction, and baseline surveys. The second visit, called session 2, occurred halfway through the study, on Day 28, for post-testing. Participant interaction data with the Amazon Echo were collected and monitored for the remaining 27 days of the study (until Day 56). During session 1, a research team member visited the participant’s home to undergo informed consent protocols, assess loneliness with the UCLA loneliness scale, and set up the Amazon Echo. The participant was then trained on how to use the device and was instructed to interact with it at least 5 times per day using commands on or like those found in the Supplementary Materials of previous research [28] for the next 27 days. The speech and hearing abilities of each participant were evaluated to confirm their capability for verbal communication with the device during the initial setup and training in session 1. On days 7, 14, and 21, reminders were sent to participants who were not meeting study interaction requirements, in an attempt to minimize researcher interaction with participants. During session 2, Day 28, the UCLA loneliness survey was administered again and participants were instructed to use their device as little or as much as they desired. On the final day of the study, Day 56, each participant was contacted and researcher access to collect new device data was relinquished. Interaction data between the participant and the Alexa device were collected for the duration of the study.

### 2.5. Data Collection

Participant interactions with the Alexa Echo device were monitored through date- and time-stamped voice records from the device’s history. During the initial device setup in Session 1, two Amazon accounts were linked to the device: a personal account and a research account. The personal participant account (which was created with the help of the researcher) was added so that the participant could continue using the device after the cessation of the study without additional researcher intervention. The participant created and/or used passwords that were not shared with the researcher to maintain confidentiality post-study. The research account was added and linked so that the team could access the Amazon Echo’s voice history for the duration of the experiment, and remotely disaffiliate from it following the conclusion of the study. Per Amazon Alexa’s legal requirement and compliance services, all voice interactions are logged into a device history file that is accessible to accounts that are linked to said device. Therefore, linking a research account enabled the team to obtain voice history data from participant interactions with the Alexa device. On the last day of the study, the interaction file was downloaded for each participant. This file contained data from participant interactions with the Echo, including the date and time of each interaction and the words spoken to it (e.g., “Alexa, what time is it?” or “Alexa, play country music”). Participants’ verbal engagement with the PVA include greetings to Alexa, such as “Hello, Alexa” or “Good morning, Alexa”; conversational questions, such as “Alexa, how old are you?”; requests for information or entertainment, such as “Alexa, is it going to rain tomorrow?”, “Alexa, play some music”, “Alexa, tell me a joke”, or “Alexa, quote a poem that would relax me”.

Using this file, each participant’s total number of words spoken was obtained through the summation of the words in every intentional command over the 4-week period. Intentional commands were defined as any interaction participants had with the device, minus any denoted as ‘not meant for Alexa’, ‘not understood by Alexa’, or ‘not stored by Alexa’ as per the device’s voice history. Hereafter, this value is referred to as the “number of intentional words”.

### 2.6. Loneliness Measurements

Loneliness was measured through a comparison of pre- and post-survey participant responses to questions from the UCLA loneliness scale. Participants completed a baseline loneliness survey during session 1 (Day 1) and were re-surveyed on Day 28. The UCLA loneliness scale is an abbreviated 8-item questionnaire that has been researched and tested as a reliable metric for analyzing loneliness [40,42]. The scale asks questions such as “I feel isolated from others”, and “I am no longer close to anyone”, which are ranked on a 5-point scale, where 1 indicates never, 2 indicates rarely, 3 indicates sometimes, 4 indicates often, and 5 indicates always feeling a certain way. Some questions, such as “I do not feel alone”, were reverse-coded using the same 5-point metric [28]. A single participant’s pre-study loneliness score was determined through an average score determinant calculated by the summation of survey answers and a division by 20 (number of questions on the UCLA scale). The same method was used to calculate a post-study loneliness score. The change in loneliness was then calculated by subtracting the post-survey loneliness score from the pre-survey loneliness score.

### 2.7. Data Analysis

The data were originally analyzed using descriptive statistics and the Shapiro–Wilks normality test to observe loneliness reduction (from baseline to 4-week loneliness survey) in the participant group (H1). Then, correlation analyses using a one-tailed Pearson’s r, predicting positive correlation, was used to observe the relationship between the number of words spoken and loneliness reduction (H2), and total PVA interaction time and loneliness reduction (H3). A mediation analysis was performed to test the increase in words spoken → more time spent → loneliness reduction mediation (H4). A partial correlation using Pearson’s r, predicting for positive correlation, was also run to control for the variability of age and gender in the participant pool.

## 3. Results

### 3.1. Participant Information

The 4-week study examined 15 individuals (N = 15) with ages falling between 77 and 96 (mean = 85.2, SD = 4.84). All participants lived independently alone, 13.33% of participants remained married and 86.67% reported being unmarried (including those single, never-married, widowed, divorced, or other). All participants were white, 73.33% were female and 26.66% were male; 67% of respondents reported having an education level less than high school, 20% completed high school, and 13% hold a college degree or higher.

### 3.2. Loneliness Reduction

**H1:**  *There will be a decrease in self-reported loneliness after the 4-week trial period.*

To determine if there was a significant loneliness reduction in the participant population over the 4-week period, descriptive statistics (Table 1) were run to analyze the loneliness baseline survey scores (mean = 2.20, SD = 0.43) and post-survey scores (mean = 1.98, SD = 0.46). The Shapiro–Wilk test, assessing assumed normality, does not indicate a reason to assume non-normal distribution (W = 0.93, *p* = 0.27). Also, an independent samples *t*-test comparing the loneliness reduction between male and female participants indicated no significant difference (t = −0.38, *p* = 0.71; male participants’ loneliness reduction: mean = 0.28, SD = 0.06; female participants’ loneliness reduction: mean = 0.20, SD = 0.40).

**Figure 1:**
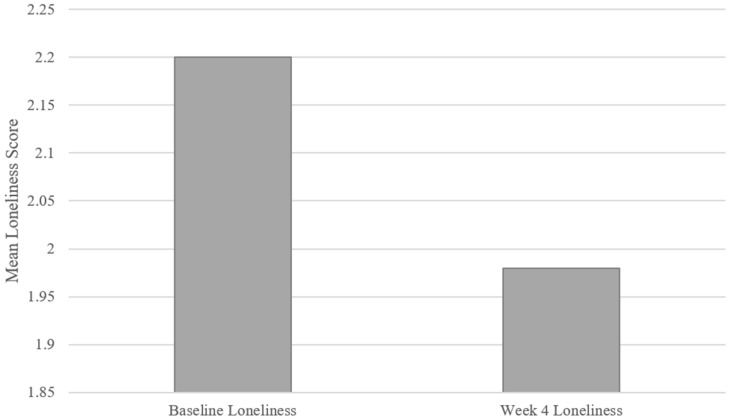
Mean loneliness scores at the baseline and week 4 loneliness measurements.

A paired sample *t*-test was run to observe the change in loneliness over the 4-week period, and the results in Figure 1 (t = 2.57, *p* < 0.05) indicate that self-reported loneliness was reduced be the end of the 4 weeks of the PVA intervention (baseline loneliness: mean = 2.20, SD = 0.43; week 4 loneliness: mean = 1.98, SD = 0.46). In sum, the results support H1, in that the test population’s loneliness significantly decreased over the course of the PVA intervention.

Before testing the relationships between intentional words spoken to Alexa, total time spent with Alexa in minutes, and loneliness reduction, descriptive statistics of these variables are summarized in Table 1.

A directional one-tailed Pearson correlation test predicting positive correlation was used to examine the correlations among intentional words spoken to the device, total time spent with the device, and loneliness reduction.

**H2:**  *There will be a positive correlation between the number of verbal engagement with a PVA and a reduction in loneliness over the 4-week period.*

There was not a significant positive correlation (at α = 0.05) between intentional words spoken and a reduction in loneliness, r(13) = −0.10 *p* = 0.64. Based on these data, there is no support for the hypothesis that the quantity of intentional words is positively correlated to loneliness reduction. Hence, H2 was not supported.

**H3:**  *Participants who spend more time interacting with their PVA device will report higher levels of loneliness reduction over the 4-week period.*

However, there was a significant, positive correlation between the total time spent with the device and loneliness reduction (r(13) = 0.46, *p* < 0.05), even after using a partial correlation controlling for gender and age, r(13) = 0.51, *p* < 0.05. Therefore, we have evidence that the total time spent interacting with Alexa is significantly positively correlated with loneliness reduction over a 4-week period, especially due to the sustained significance after controlling for age and gender. H3 was supported.

**H4:**  *There will be a “increase in verbal engagement → more interaction time → loneliness reduction” mediation path among participants over the 4-week period.*

**Figure 2:**
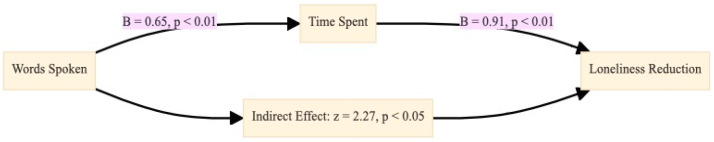
The “Words Spoken → Time Spent → Loneliness Reduction” mediation.

A Sobel test examined the “*increase in words spoken* → *more time spent* → *loneliness reduction*” mediation. The results indicated a significant indirect effect of the words spoken on loneliness reduction through the participants’ time spent with the device; z = 2.27, *p* < 0.05. Specifically, words spoken were positively correlated with time spent (B = 0.65, *p* < 0.01) and time spent was positively correlated with loneliness reduction (B = 0.91, *p* < 0.01) (see Figure 2). Therefore, H4 was supported.

## 4. Discussion

This study specifically examined the efficacy of PVA interactions over a 4-week period in mitigating feelings of loneliness. We hypothesized that prolonged engagement with a PVA device would correlate with a noteworthy decrease in self-reported loneliness (H1), theorizing that the cumulative effect of verbal engagement (H2) and the total duration of interaction (H3) would significantly contribute to this reduction. Moreover, we posited a mediation effect, where verbal engagement would lead to increased time spent with the device, which in turn would reduce loneliness (H4).

### 4.1. Findings and Implications

After a comprehensive analysis, our data revealed that participants who engaged more extensively with their PVA devices reported a significant decrease in loneliness. This supports our first and third hypotheses, affirming the therapeutic potential of PVAs in health interventions for older people [28,43]. Notably, the amount of time spent with the device emerged as a more potent predictor of loneliness reduction than the number of words spoken, suggesting the extent of engagement rather than the frequency of verbal interaction is paramount.

Consistent with previous research on the moderating role of the amount of time spent with voice AI or assistive technologies [35,36], it is possible that longer durations of interaction might have indicated a level of comfort and familiarity with the device that goes beyond simple command–response verbal interactions. This depth of engagement points to the potential of PVAs to serve not merely as functional aids but as proxies for companionship that can be especially valuable to those experiencing social isolation, which concurs with previous reports of PVA devices as verbal companions to older people [28,32].

Interestingly, the relationship between verbal interaction, as in the number of words spoken, and loneliness reduction was not direct but mediated by the amount of time spent with the PVA device. This confirms that while verbal communication is a component of PVA interaction, it is the encompassing experience of engagement as measured by total time with the device that is more influential in combating loneliness [27].

The implications of these results are significant for the gerontological field, suggesting that PVAs can serve as a form of companionship, potentially filling a void for those with limited social interactions. Also, this nuanced understanding of PVA interaction dynamics suggests a need to design PVA systems that are not only responsive to verbal commands but also proactive in fostering sustained engagement and more time interacting with the PVA device. For instance, PVAs could be programmed to initiate conversations based on user interests, maintain a dialogue over time, and even detect emotional cues to provide more empathetic interactions.

### 4.2. Limitations and Considerations for Future Research

In light of the limitations imposed by COVID-19 restrictions, which affected participant diversity and sample size, future studies could expand on this work by encompassing a broader demographic. This could enhance the applicability and generalizability of these findings, providing a more comprehensive view of the role of PVAs in alleviating loneliness across different cultures and settings. Given that our participant pool consisted solely of non-Latino whites, predominantly representative of the Midwestern nursing facility demographic [44], subsequent research should aim to include a more diverse cohort to validate and extend these findings.

This study did not include social support networks within its scope. However, future research should explore the role of these networks in the context of older individuals’ use of PVA devices. Previous research has indicated that voice-activated virtual home assistants might function as social support agents for older people [27]. Integrating social support networks into future studies could extend the present study by examining the more varied functions of PVA devices.

Moreover, while our research focused on the quantity of interaction, incorporating qualitative data on participants’ perceptions of their interactions with Alexa could offer additional insights. Longitudinal studies are also necessary to understand the sustained effects of PVA interactions on loneliness over time.

## 5. Conclusions

In summary, our exploratory study offers new insights into the use of PVA devices as a novel intervention to mitigate loneliness among older people. The key finding is that prolonged interaction with PVA devices is instrumental in reducing feelings of loneliness among older adults. Rather than the quantity of verbal commands and responses, it is the continuity of interaction that significantly influences the emotional well-being of older users.

The findings of our study have a practical significance in gerontology, indicating that PVA devices could act as companions for older people, particularly for those who experience limited social contact. This study suggests that future PVA designs should go beyond simple command responses to actively engaging users by initiating conversations tailored to their interests, sustaining interactions over time, and recognizing emotional cues for more empathetic communication, thus enhancing the companionship aspect of PVA devices.

Looking ahead, further research is warranted to build upon the preliminary findings of this study. Given its exploratory nature and the limitations posed by the homogeneity of the participant pool and the short duration of the study, future research should aim to include a more diverse and larger sample size, extend the interaction period, and incorporate mixed-method approaches to capture both quantitative and qualitative aspects of user engagement with PVA devices. Additionally, longitudinal studies could investigate the long-term effects of PVA interactions on loneliness and other related mental health outcomes in older people.

Ultimately, this study serves as a foundation for a growing field of research at the intersection of technology and gerontological care, highlighting the potential of PVA devices and artificial intelligence agents to not only assist with everyday tasks but also to contribute significantly to the emotional and social fulfillment of older adults.

## Figures and Tables

**Table 1:** Descriptive statistics of measured variables (intentional words, total time, and loneliness reduction).

Variable	N	Mean	Median	Standard Deviation	Minimum	Maximum
Intentional Words	15	2003	1851	818	386	3424
Total Time	15	49.20	38.7	28.20	8.44	95.70
Loneliness Reduction	15	0.23	0.25	0.34	−0.25	1.00

## Data Availability

The datasets presented in this article are not readily available because we do not have IRB approval to share the data. Requests to access the datasets should be directed to cyan3@unl.edu.
